# Influence of brain lesion and educational background on language tests in aphasic subjects

**DOI:** 10.1590/S1980-57642009DN20400016

**Published:** 2008

**Authors:** Ellen Cristina Siqueira Soares, Karin Zazo Ortiz

**Affiliations:** 1 Master in Sciences by Universidade Federal de São Paulo (Unifesp), São Paulo, Brazil.; 2 Post Doctor in Sciences by Universidade Federal de São Paulo (Unifesp), Departamento de Fonoaudiologia, Universidade Federal de São Paulo, São Paulo, SP, Brazil.

**Keywords:** language, aphasia, language test

## Abstract

In language assessment, several socio-demographic variables must be taken into account.

**Objectives:**

To characterize the performance of aphasic patients with different educational background on language tasks and to compare their performance to that of individuals with no language disorders.

**Methods:**

Thirty aphasic patients and 30 healthy individuals were selected. Patients were divided into two groups according to educational level: A (1–4 years) n=15 and B (5–11 years) n=15. Age ranged from 27 to 78 years. All subjects were submitted to the Montreal Toulouse language assessment protocol. The pertinent statistical tests were applied.

**Results:**

Educational level interfered in the linguistic performance of normal subjects but not in that of aphasic subjects, whose performance was influenced more by the lesion.

**Conclusions:**

The present study can contribute toward greater understanding of the influence of lesions and educational background on the language performance of aphasic subjects.

## Abstract (pt)

Para a avaliação de linguagem várias variáveis sócio-demográficas devem ser levadas em conta.

**Objetivo:**

Estudar e caracterizar o desempenho de sujeitos afásicos com diferentes escolaridades em tarefas lingüísticas e comparar o desempenho destes pacientes com indivíduos sem alteração de linguagem.

**Métodos:**

Foram selecionados 30 pacientes afásicos e 30 indivíduos, pareados com mesma idade, sexo e escolaridade. A escolaridade variou em duas faixas de acordo com o nível educacional: A (1–4 anos) n=15 e B (5–11 anos) n=15. A idade variou entre 27 e 78 anos. Todos os sujeitos foram submetidos ao protocolo de avaliação de linguagem Montreal Toulouse. Foram aplicados os testes estatísticos pertinentes.

**Resultados:**

A escolaridade interferiu no desempenho lingüístico de sujeitos normais, mas não de sujeitos afásicos, sendo para este grupo a lesão mais impactante e determinante do desempenho.

**Conclusões:**

Acreditamos que o nosso estudo contribua para a uma melhor compreensão do impacto da lesão e da escolaridade no desempenho lingüístico de sujeitos afásicos.

Language is a complex cerebral function comprising many linguistic processes. The multiple functional components of language interact to produce the end function of verbal communication.^1^

Aphasia is a loss or deficit in language function as a result of brain injury.

Language assessment in aphasic subjects seeks to measure oral and graphical production and comprehension, repetition, naming and fluency, while also assessing pragmatic and discursive functions of the language.

The investigator can devise their own tests to perform language assessment, or may elect to use previously standardized formal instruments. The tests allow performance comparisons between brain-damaged and normal subjects with the same educational background. Moreover, comparisons can also be inter-patient when the test is used longitudinally.

The present study investigated language assessment in aphasic subjects with different educational background and compared outcomes with the performance of normal subjects on a range of different language tasks. The rationale was to compare the linguistic performance of aphasic patients with healthy individuals of the same age, sex and schooling in order to ascertain:

- Which language tasks were most compromised in the aphasic group;
- Which language tasks were most affected by educational background in both aphasic and control groups.

## Methods

The present studied was approved by the UNIFESP Research Ethics Committee (protocol nº 0151/05).

Thirty subjects diagnosed with aphasia were selected.

Inclusion criteria were adult subjects, with single embolic ischemic stroke in the left hemisphere, having neurologic diagnosis and neuroimaging exam.

All the patients were assessed at the Acquired Neurologic Disturbances of Speech and Language Outpatient Unit of the Speech Therapy Department of UNIFESP-EPM in 2006.

Patients were divided into two groups based on their educational background: A (1–4 years of education) n=15 and B (5–11 years of education) n=15. Age ranged from 27 to 78 years.

All subjects were informed regarding the study and underwent language assessment using the Montreal Toulouse protocol.

The Montreal Toulouse protocol^2^ was used in the assessment. It has 22 different subtests that characterize oral and graphical production, oral and graphical comprehension, besides repetition, naming and fluency. The test enables the examiner to verify speech and language manifestations and to diagnose the type of aphasia.

The control group for the aphasic population comprised 30 subjects matched with the aphasics for age, sex and educational background, with ages ranging from 27 to 78 years. Inclusion criteria were absence of language deficits, whereas exclusion criteria were diagnosis or history of hearing, psychiatric and/or neurological disorders, based on a previously administered questionnaire. For this control group, the same criteria in relation to educational background was applied, one group comprised those with 1 to 4 years of education and a second with 5 to 11 years of education.

The Toulouse Montreal Test encompasses the following tests:

1. **Guided interview:** Subject must answer 12 open questions, some subdivided. Subtests assess both oral production and comprehension. This study scored oral comprehension.
2. **Reciting or automatisms:** The task is made up of natural series (numbers, months of the year and days of the week). One point is given for each series produced correctly.
3. **Oral comprehension:** This task involves a total of 42 items assessing oral comprehension of words, along with simple and complex phrases. Subjects provide answers through the designation task.
4. **Repetition:** The subject must repeat a total of 33 items, comprising words, non-words and phrases.
5. **Reading:** The subject has to read a total of 33 prompts such as words, non-words and phrases.
6. **Written comprehension:** 16 boards containing 6 to 4 figures are presented, and 16 written cards which the subject must read and match with the corresponding graphical prompt. There are boards representing words or simple and complex phrases.
7. **Naming:** The subject must name 31 figures.
8. **Verbal fluency:** The subject must produce the highest number of animals they can within a 60-second interval, while the investigator records the number of words produced. Each item scores 1 point. Timings are achieved using a stop-watch.
9. **Buco-facial praxis:** The subject must make 6 praxic non-verbal gestures, elicited by verbal command.
10. **Naming of body parts (oral):** The subject must understand and indicate on themselves, 8 body parts named by the investigators.
11. **Handling of objects following verbal instructions:** The subject must understand and carry out 8 commands given by the Investigator, using concrete objects (key, comb, cup, ashtray and paper). Phrase complexity steadily increases.
12. **Copying:** The subject must copy 3 words and 1 phrase.
13. **Dictation:** The subject must note down 10 words and 3 phrases dictated by the investigator.
14. **Naming of body parts (written):** The subject must understand and indicate 8 parts of the body.
15. **Written naming of actions:** The subject must graphically produce phrases referring to 6 figures arranged on 6 different boards shown by the investigator.
16. **Repetition of numbers:** The subject must repeat 10 items referring to numbers.
17. **Reading of numbers:** The subject must read 10 numbers.

All tasks in the test are awarded 1 point for each correct answer.

The data from the 17 subtests, including performance of both aphasics and controls according to educational level, underwent statistical analysis.

The Analysis of Variance test (ANOVA) was applied to compare groups among tests, adopting a 5% significance level. Multiple comparisons were calculated for those results which proved significant in order to identify these significant differences.

## Results

In our casuistic, 8 (26.6%) patients presented expressive aphasia, 10 (33.3%) receptive aphasia while 12 (40%) presented mixed aphasia.

Table 1 compares performance of aphasics and controls on tasks from the Montreal Toulouse test.

**Table 1:** Comparison between performance of aphasics and controls by mean and standard deviation on tasks from the Montreal Toulouse test, and ANOVA values.

	Group	Statistic	Educational background			ANOVA (p) values		
			1-4 years	>5 years		Group	Educational level	Group × educational level
Guided interview	Control	Mean Standard deviation N	11.85 0.38 15	11.88 0.33 15		<0.0001*	0.2634	0.2906
	Aphasic	Mean Standard deviation N	8.13 3.29 15	9.40 2.95 15				
Automatisms form	Control	Mean Standard deviation N	29.00 0.00 15	29.00 0.00 15		<0.0001*	0.9517	0.9517
	Aphasic	Mean Standard deviation N	17.47 12.39 15	17.20 11.47 15				
Automatismscontent	Control	Mean Standard deviation N	3.00 0.00 15	3.00 0.00 15		<0.0001*	0.7860	0.7860
	Aphasic	Mean Standard deviation N	1.80 1.37 15	1.67 1.29 15				
Oral comprehension	Control	Mean Standard deviation N	38.54 2.57 15	39.82 2.35 15		<0.0001*	0.3609	0.7564
	Aphasic	Mean Standard deviation N	25.93 11.85 15	28.53 10.59 15				
Repetition	Control	Mean Standard deviation N	32.46 0.97 15	32.12 0.93 15		<0.0001*	0.7508	0.8707
	Aphasic	Mean Standard deviation N	16.40 12.55 15	15.33 11.46 15				
Reading	Control	Mean Standard deviation N	31.54 1.90 15	32.41 0.80 15		<0.0001*	0.7789	0.8812
	Aphasic	Mean Standard deviation N	11.67 11.97 15	11.93 9.78 15				
Written comprehension	Control	Mean Standard deviation N	12.08 1.32 15	12.71 0.59 15		<0.0001*	0.1583	0.5699
	Aphasic	Mean Standard deviation N	6.60 3.92 15	8.07 3.83 15				
Naming	Control	Mean Standard deviation N	26.69 3.12 15	28.00 2.37 15		<0.0001*	0.2940	0.7100
	Aphasic	Mean Standard deviation N	11.07 10.26 15	13.80 9.81 15				
Verbalfluency	Control	Mean Standard deviation N	13.92 6.45 15	21.24 7.66 15		<0.0001*	0.0087*	0.1054
	Aphasic	Mean Standard deviation N	4.53 5.49 15	6.33 5.83 15				
Buco-facialpraxias	Control	Mean Standard deviation N	5.77 0.44 15	5.82 0.53 15		<0.0001*	0.9353	0.9353
	Aphasic	Mean Standard deviation N	4.13 1.85 15	4.13 1.64 15				
Naming ofbody parts(oral)	Control	Mean Standard deviation N	8.00 0.00 15	7.88 0.49 15		<0.0001*	0.5162	0.6978
	Aphasic	Mean Standard deviation N	6.53 2.26 15	6.07 2.55 15				
Handling ofobjects	Control	Mean Standard deviation N	7.92 0.28 15	7.88 0.33 15		<0.0001*	0.7119	0.6425
	Aphasic	Mean Standard deviation N	4.64 2.27 15	5.00 2.33 15				
Copying	Control	Mean Standard deviation N	3.92 0.28 15	4.00 0.00 15		<0.0001*	0.8870	0.8870
	Aphasic	Mean Standard deviation N	1.53 1.60 15	1.53 1.30 15				
Dictation	Control	Mean Standard deviation N	8.62 3.10 15	11.94 1.84 15		<0.0001*	0.0326*	0.0479*
	Aphasic	Mean Standard deviation N	2.73 3.75 15	2.87 3.14 15				
Naming of body parts (written)	Control	Mean Standard deviation N	7.77 0.60 15	7.88 0.33 15		0.0007*	0.4408	0.5529
	Aphasic	Mean Standard deviation N	3.60 3.29 15	4.47 3.52 15				
Written naming-actions	Control	Mean Standard deviation N	5.08 1.04 15	5.94 0.24 15		<0.0001*	0.0324*	0.9976
	Aphasic	Mean Standard deviation N	0.80 1.78 15	1.67 2.26 15				
Repetition - numbers	Control	Mean Standard deviation N	10 0 15	10 0 15		<0.0001*	0.6438	0.6438
	Aphasic	Mean Standard deviation N	5.53 4.09 15	4.87 3.72 15				
Reading - numbers	Control	Mean Standard deviation N	9.23 1.24 15	9.88 0.33 15		<0.0001*	0.3889	0.9921
	Aphasic	Mean Standard deviation N	4.47 3.74 15	5.13 4.34 15				

This table shows that the group effect compared the performance of aphasics and controls whereas the educational effect compared educational background in each group – aphasic and control groups. Concerning the Group effect vs. educational background, comparisons were made between groups according to educational profile.

For verbal fluency and dictation tasks, we observed that in terms of group effect, aphasic subjects presented fewer responses than controls at both levels of education. For the educational background effect, the control group presented fewer responses in the 1–4 year education bracket, yet no such difference was seen among aphasics with different educational background.

Concerning written naming of action tasks, we observed that in terms of group effect, aphasic subjects always presented fewer responses than controls. For the educational background effect, the control group presented fewer responses in the 1–4 year education bracket, yet no such difference was seen among aphasics with different educational background.

With regard to the remaining tasks in terms of group effect, aphasic subjects presented fewer responses for both educational bands.

## Discussion

The aphasic subjects presented lesions at different sites within the left hemisphere, although all had suffered a single embolic ischemic stroke. It is known that there is no strict correlation between the injured area of the central nervous system and the type of aphasia,^3,4^ a fact corroborated by the present study. Studies in the literature have also indicated that, in the majority of right-handed patients, language processing takes place predominantly in the left hemisphere.^4^

In terms of characterizing the sample distribution, we observed that the groups of patients with expressive, receptive and mixed aphasia were similar in number.

Table 1 shows no difference comparing performance of aphasic subjects on a range of tasks from the Montreal Toulouse protocol, with regard to educational background.

First, we can observe that the comparison of the performance of aphasic subjects and controls of the same age, sex and schooling, yielded statistically significant differences across all tasks in the Montreal Toulouse protocol, showing this to be an effective tool for assessing language compromise in aphasic patients.

The control group presented statistically significant difference in performance with increased educational-level on verbal fluency, dictation and written naming of action tasks. Drawing on these results, we may infer that educational level modifies the performance profile in subjects on these tasks, most likely due to the development of a meta-language and skills acquired over the course of the knowledge-building process during school and academic learning. These differences were not observed in aphasic patients. This data will be discussed below.

Several models in cognitive neuropsychology explain some of the oral and graphical language processes.^5-7^ These models are commonly used for studying single clinical cases, in which specific changes to some linguistic processing components can be better identified. The models can ensure greater understanding of the disorder presented by the patient, while also guaranteeing improved treatment planning. Population studies provide a profile of the most prevalent manifestations but do not specify which language processes are impaired. This can only be achieved using individual case studies. In the present study, statistically significant differences were observed between aphasic and control groups for all language tasks of the Montreal Toulouse test.

Language tasks are known to entail complex involvement of many areas of the brain. Our results allow us to hypothesize that a brain lesion in an aphasic patient may correlate with poorer performance in language tasks.

Our study demonstrated that all oral comprehension tasks in the Montreal Toulouse Test, word comprehension, simple phrases (subject, verb and complement) and complex phrases (coordinated, subordinated and passive voice utterances), allowed us to differentiate aphasic subjects from those without brain injury.

Aphasic subjects can present a range of errors in oral production tasks, whereas educated subjects without brain injury are able to correctly repeat words, pseudowords and non-words.^8,9^ The statistically significant difference found between the group of aphasics and controls can be explained upon verifying the manifestations presented by aphasic subjects. Examination of errors committed by aphasic subjects reveals that these errors must have occurred due to failure in phonologic access or failure in motor programming of speech, followed by, to a lesser extent, failures in lexical and semantic access.

Comparison between the performance of aphasic subjects and controls regarding verbal fluency tasks highlights the failure of aphasic patients in accessing the lexicon. Moreover, this access difficulty occurred independently of educational level. This difficulty accessing the lexical buffer is linked to the brain lesion, and thus aphasics present poorer performance than controls. In fact, anomia is the most frequent manifestation in aphasias.^1^ It is known that large anterior and posterior areas seem to be involved in lexical access^10^ making aphasic subjects vulnerable to this manifestation.

Concerning writing, cognitive writing models assume the existence of different storage and procedure memories involved in this function. These models assist in analyzing performance of patients following neurologic impairment. Paragraphies are manifestations in writing which are seen in aphasic pictures. This type of manifestation was observed in all tasks of the present study such as copying, graphical naming and writing by dictation. The writing process, including copying, writing by dictation and naming, involves the cognitive domains of attention, cognitive flexibility, memory, visual processing and praxias, rendering it more vulnerable in the event of a neurologic injury.

In the dictation task, after hearing a prompt the subject should recognize the words as a sequence of syllables of the language, or as a whole word in the lexical recognition. The stimulus may undergo three types of processing: lexical, syllabic or phonological. Information can be processed by lexical semantic access or can undergo phonological-orthographic conversion, with information flowing to the graphosyllabic output register, having two options of processing: information can proceed to the orthographical output and on to the letter-alphabetic output, or can go directly to the latter (syllabic segmentation processing). Another information processing mechanism is whereby phonosyllabic input register information goes to the literal input register, and from here letters are matched in the alphabetic output register (literal segmentation processing). After any of the three processes outlined above, the information is processed in the graphemic buffer, proceeding to the allographic buffer where programming and execution of chiroarticulatory hand movements finally takes place resulting in writing.^11^ Regardless of the kind of processing used by aphasic subjects in the dictation task, their performance was statistically lower when compared to controls (Table 1). This appears to indicate distinct alterations in language processing of writing compared with performance on other tasks of the test in the aphasic population. Taken together, the data highlight that the aphasic group indeed presented specific alteration in writing.

In reading tasks, graphical symbols are processed within the occipital, inferior-temporal and parietal lobes.^12-14^ Several cortical and subcortical areas in the left hemisphere are involved in reading processing explaining the frequent occurrence of central dyslexias co-occurring with aphasias.^15,16^ The reading aloud tasks performed in this study from the Montreal Toulouse test are composed of stimuli able to assess the lexical and phonological routes.^11^ Aphasic subjects presented errors in all cues, demonstrating lower performance than controls.

Written comprehension tasks entail matching written content with a given figure. In this case, the subject has to perform visual analysis both of written content and the corresponding figure. In the event that the patient presents visual difficulty or when the figure is unclear, the subject may fail on the subtest due to a failure in visual analysis prior to graphical processing of the written information. Although this possibility existed in the present study, we observed that aphasic subjects presented difficulties in matching the written content with the appropriate figure, most likely owing to failure in graphical comprehension or arising from difficulties in carrying out integration of the written content with the pictograph. Aphasic subjects presented deficits in graphical comprehension ranging from mild to severe, leading to worse performances compared to controls.

Concerning buco-facial praxias, a statistically significant difference was evidenced between aphasic and control groups. In fact, aphasic patients presenting verbal and non-verbal praxic alterations are frequently seen in clinical practice. Dronkers^17^ found that 48% of patients presenting brain injury also had verbal or non-verbal apraxia. Moreover, phonemic paraphasias are manifestations which may stem from verbal apraxia. The high rate of phonemic paraphasias in expressive and mixed aphasia groups, in conjunction with the non-verbal praxic alterations in these two groups, lead us to hypothesize on the co-occurrence of verbal and non-verbal apraxia in some patients of this study.

We can argue that no difference was evident since the factor determining performance was language impairment resulting from brain injury and not schooling. Thus, lesions were the strongest determinant of language behavior in aphasics, preventing the stronger language performance in more highly educated individuals commonly seen in healthy populations.

In fact, several studies^18-20^ have reported no performance difference in brain-damaged subjects on neuropsychological tests based on level of schooling. Our findings revealed the same type of effect, albeit in relation to language, that is, higher-education in brain-damaged subjects was not associated with better performance in language tests. This is most likely because the injury compromised language abilities thereby causing aphasic groups with different educational level to perform similarly in terms of language processing.

We conclude that differences were observed across all language tasks of the Montreal Toulouse test upon comparison of aphasic patients with controls. Although differences were found in language performance on some tasks by the control group according to schooling, this did not occur in the group of aphasic patients, in which the lesion itself appeared to have greater influence. Finally, worse performance was observed in aphasics on verbal fluency, dictation and written naming of action tasks.

The authors believe the present study contributes toward understanding the role played by lesions and schooling in aphasic patients, proving valuable to professionals involved in assessment and rehabilitation of this patient population, especially in countries with large social cultural diversity.
